# Occurrence of Enterococci in the Process of Artisanal Cheesemaking and Their Antimicrobial Resistance

**DOI:** 10.3390/life14070890

**Published:** 2024-07-18

**Authors:** Zuzana Hanzelová, Eva Dudriková, Viera Lovayová, Jana Výrostková, Ivana Regecová, František Zigo, Klára Bartáková

**Affiliations:** 1 Department of Food Hygiene, Technology and Safety, The University of Veterinary Medicine and Pharmacy in Košice, 041 81 Košice, Slovakia; zuzana.hanzelova@student.uvlf.sk (Z.H.); jana.vyrostkova@uvlf.sk (J.V.); ivana.regecova@uvlf.sk (I.R.); 2 Department of Medical and Clinical Microbiology, Faculty of Medicine, Pavol Jozef Šafárik University in Košice, 040 11 Kosice, Slovakia; viera.lovayova@upjs.sk; 3 Department of Animal Nutrition and Husbandry, The University of Veterinary Medicine and Pharmacy in Košice, 041 81 Košice, Slovakia; frantisek.zigo@uvlf.sk; 4 Department of Animal Origin Food & Gastronomic Sciences, University of Veterinary Sciences Brno, 612 42 Brno, Czech Republic; bartakovak@vfu.cz

**Keywords:** enterococcal isolates, processing environment, MALDI-TOF MS, PCR, silent genes

## Abstract

Enterococci are a group of microorganisms that have a controversial position from some scientific points of view. The species of the greatest clinical importance are *E. faecalis* and *E. faecium*, which are common agents of nosocomial infections. However, enterococci also have important applications in the dairy industry, as they are used as non-starter lactic acid bacteria (NSLAB) in a variety of cheeses, especially artisanal cheeses. The aim of this study was to determine the presence of representatives from the *Enterococcus* genus using PCR and MALDI-TOF MS methods on samples of raw milk, processing environment swabs, and cheese from four different artisanal dairy plants in Slovakia. Among the 136 isolates of enterococci, 9 species of genus *Enterococci* (*E. faecalis*, *E. faecium*, *E. durans*, *E. devriesi*, *E. hirae*, *E. italicus*, *E. casseliflavus*, *E. malodoratus*, and *E. gallinarum*) were identified and were tested for their antimicrobial resistance (AMR) to 8 antibiotics (amoxicillin, penicillin, ampicillin, erythromycin, levofloxacin, vancomycin, rifampicin, and tetracycline); most of them were resistant to rifampicin (35.3%), ampicillin (22.8%), and tetracycline (19.9%). A PCR analysis of *vanA* (4.41%) and *tetM* (14.71%) revealed that antimicrobial resistance genes were present in not only phenotypic resistant isolates of enterococci but also susceptible isolates. The investigation of antimicrobial resistance in enterococci during the cheesemaking process can be a source of valuable information for public health in the concept of “One Health”.

## 1. Introduction

Enterococci can be concisely described as Gram-positive ovoid cocci occurring singly or in pairs, both in short chains and in clusters [1]. They are non-sporular and they can produce yellow pigment. Enterococci are catalase-negative, but when cultured on blood media, they can show a positive catalase reaction [1,2].

Enterococci occur and grow in a variety of cheeses, especially those made from raw or pasteurized goat’s, ewe’s, or cow’s milk. In these types of milk, the most commonly detected *Enterococcus* spp. are *E. faecium*, *E. faecalis*, *E. hirae*, and *E. durans* [3]. Within this genus, there is a specific group of bacteria that can play a role in cheesemaking; these representatives are generally referred to as controversial microorganisms due to their simultaneous positive (producing bioactive peptides) and negative (biogenic amine producer) influences in the process [3,4,5]. Enterococci have an important role in the production of certain types of cheese and fermented sausages produced in the Mediterranean basin. Through their lipolytic and proteolytic activity, they contribute to the development of desirable organoleptic characteristics [6,7]. In many artisanal cheeses, enterococci are part of the non-starter lactic acid bacteria, and they have, in some instances, been used as components of experimental starter cultures [8].

The impact of the enterococcal microbiota of artisanal cheeses on the health of their hosts is still the object of debate, despite the wealth of knowledge gathered in recent years on their presence, technological properties, potential health benefits, antibiotic resistance, and carriage of virulence factors [9]. There are possible risks associated with each method of cheese production, specifically through the transfer of microorganisms that may pose a risk to the health of the final consumer. Therefore, it is necessary to ensure adequate hygiene conditions from the time when the milk is obtained to the time when the product is unpacked by the consumer. The European Union has established a maximum limit for the presence of coliforms and *Escherichia coli* in some dairy products, both of which are considered indicators of hygiene, while no limit has been set for enterococci. *Enterococcus* spp. do not have a generally recognized as safe (GRAS) status nor are they included on the qualified presumption of safety (QPS) list [10,11,12].

Antibiotic resistance is a global health challenge, involving the transfer of bacteria and genes between humans, animals, and the environment. A set of genetic determinants allows enterococci to colonize their host efficiently, and they can exchange genes with other bacteria, promoting their adaptation to the seemingly inhospitable conditions they face [13]. The acquisition of antimicrobial resistance during the development of enterococci is one of the factors that explain their success as nosocomial pathogens [14]. Enterococci demonstrate these abilities to acquire antimicrobial resistance through the exchange of resistance-coding genes transmitted by transposons and plasmids. Plasmids are abundant in enterococci and entail another important contribution to their genomic plasticity [8,15]. Species of the genus *Enterococcus* are naturally resistant to cephalosporins and low concentrations of aminoglycosides, sulphonamides, clindamycin, quinupristine, dalfopristin, and most beta-lactams. In addition, *E. casseliflavus* and *E. gallinarum* have natural resistance to low vancomycin concentrations [16]. Acquired enterococcal resistance includes resistance to chloramphenicol, erythromycin, tetracycline, fluoroquinolones, glycopeptides, and high concentrations of clindamycin, aminoglycosides, and beta-lactams [13,16]. Despite the profusion of studies on antibiotic resistance and its genetic determinants of clinical enterococci, few studies are available that focus on enterococcal species carried by traditional cheeses [8].

Based on the above, the aim of our study was to determine the occurrence of *Enterococcus* spp. and identify them in raw bulk milk, milk after pasteurization, the environment of cheesemaking, and cheese made from pasteurized milk in Slovakia and subsequently detect antimicrobial resistance and the presence of antimicrobial resistance genes.

In addition, most studies have addressed the occurrence of enterococci and their antimicrobial resistance in raw milk or cheeses made from raw milk, but few studies are focused on the cheesemaking environment and the occurrence of enterococci in cheeses, even though they are made from pasteurized milk. Also, evaluation of the two standard systems for assessing AMR may show differences between them. This study may contribute to the acquisition of knowledge in this area.

## 2. Materials and Methods

### 2.1. Isolation and Identification of Strain

**Figure 1:**
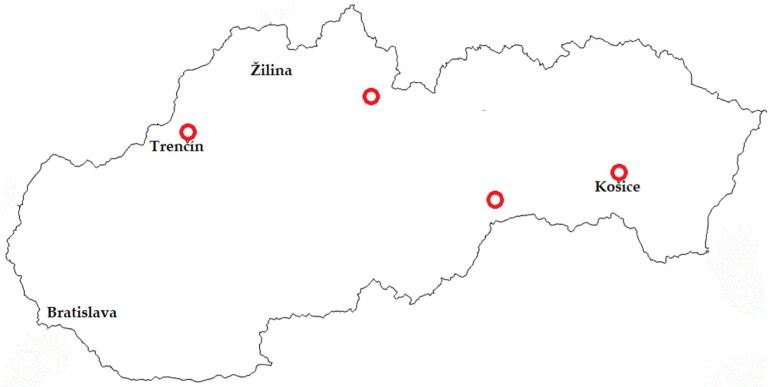
Map of Slovakia with places (red circles), where all samples were taken for the study.

A total of 127 samples were taken disposably from four different dairy plants in Slovakia (Figure 1) that are focused only on artisanal cheesemaking. The samples (Table S1) included raw bulk milk (*n* = 14), milk after pasteurization (*n* = 14), swabs from the cheesemaking environment (*n* = 75), and produced cheeses (*n* = 24). Cheeses are made from pasteurized milk through the addition of starter cultures and it is possible to buy them in the local shop as well as in supermarkets in Slovakia.

The swab samples, samples of raw milk, and pasteurized milk were taken in the dairy plants. Cheeses that had aged for a maximum of three weeks were selected, and samples were taken from dairy plants immediately after packaging. The raw bulk milk and milk after pasteurization was taken in volumes of 500 mL per sample. The moistened swabs were taken from various places (Table S2) from the areas where it was possible to take swabs using sterile swab templates with a capacity of 100 cm^2^ according to ISO 18593 [17]. The other swabs were taken according to availability (molds, hands, switches, knives) without using the corresponding area size. The cheeses taken were packaged by producers in a quantity of at least 150 g per sample. Milk samples were placed in sterile sample containers and swabs in sterile tubes with 0.1% (*wt*/*vol*) peptone water, and all samples were transported at 4 °C without the addition of preservatives to the laboratory at the University of Veterinary Medicine and Pharmacy in Košice for analysis. Within 4 h of collection, all samples were subjected to microbiological analysis.

All the samples underwent decimal dilution according to ISO 6887-5 [18]. Then, the samples in volume 0.1 mL were streaked on Slanetz and Bartley agar (Merck, Germany) plates and incubated at 37 °C for 24–48 h. After incubation, the bacterial count was evaluated. Subsequently, based on characteristic appearance and typical colonies in numbers of three per plate were taken and purified on Brain Heart Infusion Agar (Merck, Germany) and stored at −18 °C for further investigation.

The DNA was extracted from the 156 enterococcal strains according to Hein et al. [19]. PCR was used to identify the genus of *Enterococcus* according to Ke et al. [20] and Martineau et al. [21]. The primers derived from the *tuf* gene were used to obtain a specific sequence for the genus *Enterococcus* spp. Each primer was synthesized in Genomed (Table 1; Warszawa, Poland). The PCR protocol was optimized as follows: initial denaturation at 95 °C for 13 min, followed by 30 cycles (denaturation at 95 °C for 20 s, annealing at 52 °C for 30 s, and extension at 72 °C for 2 min). The final extension was performed at 72 °C for 10 min. The HotFirepol^®^ Mastermix (Solis BioDyne, Tart, Estonia) was used in the PCR. The PCR products were visualized in a 1.5% agarose gel with Goldview™ nucleic acid staining (Beijing SBS Genetech Co., Ltd., Beijing, China) by using the MiniBIS Pro^®^ (DNR Bio-Imaging system Ltd., Neve Yamin, Israel).

After PCR identification, the 136 isolates belonging to the *Enterococcus* genus (Figure S2) were streaked on Columbia Blood Agar (Merck, Darmstadt, Germany) to prepare for MALDI-TOF MS examination and incubated at 37 °C for 24 h. To make the analysis as accurate as possible, individual samples were prepared via an extraction procedure using ethanol and formic acid [23]. Then, the colonies were used for identification using MALDI-TOF MS (Bruker Daltonics, Billerica, MA, USA).

### 2.2. Antimicrobial Susceptibility Testing

The identified *Enterococcus* spp. isolates were tested for antibiotic susceptibility using the disc diffusion method (DDM) according to the procedure described in the CLSI document [24]. Eight antibiotics were tested: amoxicillin (AML, 25 µg), penicillin (P, 10 µg), ampicillin (AMP, 10 µg), erythromycin (E, 10 µg), levofloxacin (LEV, 5 µg), vancomycin (VAN, 30 µg), rifampicin (RD, 5 µg), and tetracycline (TET, 30 µg). Antibiotics were selected based on the most common antimicrobial agents used in veterinary and human medicine in Slovakia for treatment [25,26]. *Enterococcus faecalis* ATCC 29212 was used as a reference strain. The results were evaluated according to two systems, namely the European Committee on Antimicrobial Susceptibility Testing [27] and the Clinical and Laboratory Standards Institute guidelines [24]. The disks used for susceptibility testing were manufactured by HIMEDIA, Mumbai, India. The diameters of the zone of inhibition were recorded in millimeters (mm) and interpreted as susceptible or resistant.

### 2.3. Detection of Antimicrobial Resistant Genes (ARG)

Genes that can confer antimicrobial resistance (AMR) to glycopeptide, *vanA*, and tetracycline, *tetM*, were detected in a PCR using the gene-specific primers shown in Table 2. The PCR protocol was optimized as follows: initial denaturation at 95 °C for 13 min, followed by 30 cycles of denaturation at 95 °C for 20 s, annealing at different temperatures depending on the gene for 30 s (Table 2), and extension at 72 °C for 2 min. The final extension was performed at 72 °C for 10 min. PCR amplicons were run on 1.5% agarose gel. The expected sizes of PCR products differed for each gene (Table 2).

### 2.4. Data Analysis

Data were entered, cleaned, and validated in a Microsoft TM Excel spreadsheet (MS Office Excel^®^ 2021, Wanchai, Hong Kong). The average number of enterococci in the raw milk and cheese was recalculated to log_10_ transformation. The distribution of each species in the raw milk was determined by calculating the percentage of each species out of the total number of enterococci isolated. The PCR results (positive or negative) were reference variables for descriptive analyses. Univariate analyses were conducted for descriptive statistics and the data were presented as percentages.

## 3. Results

### 3.1. Isolation and Enumeration of Enterococci

Enterococci were presented in 52 (40.94%) of all 127 tested samples. In total, 11 (78.57%) of 14 raw bulk milk samples, 31 (41.33%) of 75 environmental samples, and 10 (41.67%) of 24 cheese samples harboring the presence of enterococci. The average enumerations of enterococci in raw milk and cheese were 3.38 log_10_ ± 0.39 CFU/mL and 3.88 log_10_ ± 0.79 CFU/g, respectively (Table 3). In all the samples of milk, after batch pasteurization at a low temperature, no enterococci were detected. The milk prepared via pasteurization at each dairy plant was used for cheesemaking.

The samples from the environment were divided into nine groups (Table S2). The highest number of positive samples were from the samples from ripening rooms, work aprons, and vats for cheesemaking. Only one swab was evaluated for the work apron; this is because the workers of the examined dairy plants use washable work aprons. No enterococci were detected in swabs from pasteurization equipment. The evaluation of enterococcal counts in the environment was not included in the study, considering it was not possible to take samples with the same area from some types of swabs, such as, for instance, hands, molds, handles, and switches, but the presence of enterococci in these parts was of interest to our investigation.

In the raw milk, environment, and cheese samples, experimental analysis confirmed the presence of seven, five, and five species of enterococci, respectively (Table 4). Only in raw milk were *E. malodoratus* and *E. hirae* found, and only in cheeses was *E. gallinarum* detected. Overall, the most common species were *E. faecalis* (38.97%) and *E. faecium* (34.56%), but only one isolate of *E. faecium* was detected in raw milk. The majority of *E. faecium* isolates were found in the environment and cheeses.

### 3.2. Antimicrobial Susceptibility Test Results for Enterococci

Antimicrobial susceptibility results were evaluated separately according to EUCAST 2023 and CLSI 2023 documents because of the differences between these two systems. Evaluation using the EUCAST document was possible for only four of the selected antimicrobial agents (AML, AMP, VAN, and LEV). The zone diameter breaking points defined by EUCAST are different compared to values defined by CLSI. The results according to EUCAST are presented in Table 5 and Table 6. 

No similarities can be found in the evaluation results between EUCAST and CLSI documents (Table 7 and Table 8) because the final interpretation guidelines are different for all agents except amoxicillin. In particular, the percentage of resistant isolates is lower according to the evaluation of AMR according to EUCAST compared to CLSI for similar agents. Evaluation by CLSI (Table 7) showed that all the isolates of enterococci were only fully susceptible to amoxicillin. For penicillin, erythromycin, vancomycin, levofloxacin, and tetracycline, the rates of resistance among isolates were in the range of 2.9–19.9%. Higher resistance to rifampicin (35.3%) and ampicillin (22.8%) were observed. Isolates from raw milk showed the highest resistance to ampicillin (56.8%) and rifampicin (34.1%) and isolates from the environment showed the highest resistance to rifampicin (29.2%). Isolates from cheeses showed the highest resistance to rifampicin (43.2%) and vancomycin (31.8%). The distribution of enterococci by species showed that in all isolates of *E. faecalis*, the highest resistance to rifampicin (62.3%) and vancomycin (33.9%) was observed. Furthermore, in isolates of *E. faecium*, the highest resistance to erythromycin (21.3%) was observed. The resistance levels of other species are listed in Table 8.

Multidrug resistance (MDR) is defined as resistance to three or more classes of antibiotics. In this study, overall, thirty-five isolates evaluated using the CLSI 2023 document were multidrug resistant. Four isolates (milk and cheeses) were resistant to five antibiotics. Isolates from the environment were the most resistant to three antibiotics; however, this group contained the most isolates that were not resistant to any antibiotics. The most resistant isolates were *E. faecalis*, all of which were resistant to at least one to five antibiotics, except one isolate. On the other hand, thirty-two isolates of *E. faecium* were reported as sensitive to all the tested antibiotics. Overall, only five isolates from the other species of enterococci, including *E. devriesi* (2), *E. durae* (1), *E. hirae* (1), and *E. gallinarum* (1), were multidrug resistant to a maximum of four antibiotics.

### 3.3. Detection of Resistance Genes among All Isolates Genus Enterococcus

The evaluation of enterococci safety through the in vitro expression of virulence traits does not always reflect the real hazard in these microorganisms because of the presence of silent genes, which could potentially be activated by environmental conditions, thus transforming these bacteria into pathogens or enhancing their pathogenicity [30]. The isolates of enterococci were tested for the resistance genes *vanA* (Figure S2A) and *tetM* (Figure S2B). We focused on only two genes, but they were studied not only in isolates with detected resistance but also in those that were evaluated as sensitive to vancomycin and tetracycline.

Among all the identified isolates that belonged to the *Enterococcus* genus, there were five *E. faecalis* isolates and one *E. devriesi* isolate with the resistance gene *vanA*. However, only two isolates with *vanA* were evaluated as resistant according to CLSI, while four isolates with *vanA* were susceptible.

Overall, 20 isolates of enterococci carried *tetM*, with most of them being *E. faecalis* (12) and the others being *E. durans* (3), *E. devriesi* (1), *E. italicus* (3), and *E. gallinarum* (1). There were no *E. faecium* isolates with identified *tetM* genes. According to CLSI, out of the 20 isolates with confirmed *tetM*, 14 and 6 were evaluated as resistant and susceptible, respectively. The occurrence of the gene in all enterococcal isolates, regardless of phenotypic manifestation, was 14.7%. Multidrug resistance was detected in ten isolates with a confirmed *tetM* gene; nine isolates were *E. faecalis* and one isolate was *E. gallinarum*. No enterococcal isolate carried both genes studied. Raw milk samples contained the highest occurrence of both *vanA* (6.8%) and *tetM* (29.6%). Enterococcal isolates from the environment of cheesemaking revealed two isolates with the *vanA* gene and four with the *tetM* gene. Isolates from the environment, which included both studied genes originated in molds, aprons, and tables for draining.

## 4. Discussion

This study indicates that the occurrence of representatives from the *Enterococcus* genus can be expected in each stage of cheesemaking without exception, including their presence in raw milk, which is the basic raw material for cheesemaking. In spite of the fact that all the milk used for cheesemaking was pasteurized before processing, enterococci were found in the final products. The occurrence of enterococci was in 78.6% of the raw milk samples, which is higher than that found in the study from Nigeria (12%) [31]. The percentage of positive samples of enterococci in a study in Iraq [32] was 31% in raw milk. In the studies in Slovakia, the number of enterococci determined in the raw bulk milk ranged from 3.11 to 3.46 log_10_ CFU/mL and 3.32 to 4.51 log_10_ CFU/mL in the samples from the tank. The average number in raw milk was 3.92 log_10_ CFU/mL. Authors of the study noted the presence of enterococci in milk after pasteurization in the number of less than 1 log_10_ CFU/mL [33]. These bacterial counts are only slightly higher than in our findings except for the absence of enterococci in pasteurized milk in our case.

According to our study, the species that were the most commonly isolated from raw milk were *E. faecalis* (63.64%), *E. hirae* (11.36%), *E. durans* (9.09%), *E. devriesi* (6.82%), and *E. malodoratus* (4.54%). *E. faecium* was detected in only one case. Similar results were observed by Bouymajane et al. [34] in their study, where *E. faecalis* (64.7%), *E. durans* (11.8%), and *E. hirae* (5.9%) were the most common enterococcal isolates. Another study carried out in Australia reported a similar distribution of enterococci in raw milk, with a majority of isolates being *E. faecalis* (74.3%) and a minority of isolates being *E. faecium* (8.6%) [35]. The presence of *Enterococcus* spp. in food in general and particularly in consumed raw milk increases the risks to public health. Infants, the elderly, and immunodeficient patients are the populations most vulnerable to poisoning [34]. Therefore, it is worth considering the safety of the consumption of raw milk and raw milk products by these groups.

In general, it is known that there are many sources of enterococci in different types of environments. In our investigation, we predicted that we would find enterococcal representatives in the cheesemaking environment. Many studies focus on hospital environments and are oriented toward clinical isolates. In our study, swab samples were taken from the cheesemaking environment only after regular disinfection in dairy plants by focusing on places of possible food contamination. The occurrence of enterococci in the environment of cheesemaking was in 41.33% of tested samples, and the isolated species were *E. faecium* (50.0%), *E. faecalis* (25%), *E. durans* (14.58%), *E. italicus* (8.33), and *E. casseliflavus* (2.08%). This suggests that the enterococcal diversity in the environment was lower than that in raw milk. The greatest difference was in the presence of *E. faecium*, which had a similar presence in the environment and cheese (50.0%) and a lower presence in raw milk (2.27%). Gaglio et al. [36] described their study as the first report on the presence, antimicrobial resistance, and virulence genes of enterococci isolated during different steps of cheesemaking in Sicilia, including raw milk, equipment surfaces, and cheese. They worked with a total of 40 enterococcal isolates. There were 13 samples from the environment (only wooden vat surfaces), mostly including *E. faecalis* (53.85%) and *E. faecium* (38.46%), and only 1 isolate of *E. gallinarum*. As already mentioned, in many non-clinical contexts, the presence of enterococci is presumptively underestimated because most studies focus only on clinical isolates [37].

From a food safety perspective, cheeses are a potential source of bacteria, which can affect public health because of the presence of resistance genes and virulence factors. The third group investigated for the occurrence of enterococci in this study was cheeses produced from milk after heat treatment according to the relevant legislation of Slovakia. There are currently no legislative requirements for the monitoring of enterococcal presence and numbers in cheeses made from raw or pasteurized milk. A higher quantity of them in the cheeses, especially those made from pasteurized milk, may be a sign of poorer hygienic conditions, mainly if they originate from the processing environment. Enterococci may be present in large numbers in dairy products (up to 8 log_10_ CFU/g) [8]. In a study from Iran [38], the prevalence of enterococci ranged from 4.99 to 5.041 log_10_ CFU/g and 3.04 to 3.99 log_10_ CFU/g in Urmia and Tabriz cheese samples, respectively. Most studies focus attention on determining the numbers of enterococci in cheeses made from raw milk. For instance, a study with typical cheese from Slovakia, lump sheep cheese made from unpasteurized milk, showed that the total enterococcal count reached 3.93 log_10_ CFU/g ± 1.98, on average [3,5]. In our study, the number of enterococci determined in the cheeses ranged from 2.78 log_10_ CFU/g to 5.48 log_10_ CFU/g, and the total enterococcal count reached 3.88 log_10_ CFU/g ± 0.79, on average.

Enterococci were presented in 41.67% of all tested cheese samples. The isolated species were *E. faecium* (50.0%), *E. faecalis* (29.55%), *E. devriesi* (11.36%), *E. durans* (6.82%), and *E. gallinarum* (2.27%). The percentages of *E. faecalis* and *E. faecium* in cheeses were very similar to those in the environment. The most frequent species detected in artisanal cheeses produced with ewe’s, goat’s, buffalo’s, and cow’s milk, both pasteurized and raw, were also *E. faecalis* and *E. faecium* [39,40]. Studies show that, in cheeses produced from cow’s pasteurized milk ripened for up to three weeks, it is possible to find other enterococcal species such as *E. hirae*, *E. ratti*, and *E. villorum*. Similar results compared to our research were observed in studies from the UK and Italy in cheeses such as Stilton, Talegio, and Toma Piemontese, where the dominant representatives of enterococci were *E. faecium*, *E. faecalis*, and *E. durans* [8,41,42].

In this study, antimicrobial resistance was investigated, and the results show phenotypic variability across individual stages of cheesemaking. Overall, isolates of enterococci were the most resistant to rifampicin (35.3%), both in the environment (29.2%) and in cheeses (43.2%). Rifampicin is a member of the antibiotic group referred to as reserve antibiotics according to the ESAC Net 2021 report [25]. It is commonly used in the hospital sector in Slovakia, and its consumption has been increasing for the last ten years. Importantly, rifampicin is used to treat tuberculosis in human medicine. Jahansepas et al. [38] investigated resistance to rifampicin in enterococcal isolates in two groups, namely clinical isolates and traditional cheeses. The results showed a high level of resistance in both groups, with 76.25% resistance in clinical isolates and 79.17% resistance in cheeses. The increase in resistance to reserve antibiotics is a globally relevant threat to public health.

In enterococcal isolates from raw milk, the greatest resistance was to ampicillin (56.8%), rifampicin (34.1%), and tetracycline (29.6%). According to ESVAC 2023 [26], antibiotic use in Slovakia in food-producing animals is primarily based on the penicillin group and tetracycline antibiotics, which are mainly used for treatment in veterinary medicine. The trend of antibiotic consumption has been stable in Slovakia for the last four years. It is important to consider country-specific data and treatment patterns when assessing AMR because, while AMR is a global problem that evolves over time, trends in antibiotic use are country-specific, and resistance outcomes may vary from country to country. A study in Slovakia investigated AMR in cow’s milk; the *Enterococcus* genus was detected in 3.08% of isolates, but AMR was found to be more predominant in other species of bacteria [43]. According to Hammad et al. [44], *Enterococcus* spp. isolates from raw cow’s milk were resistant to erythromycin (87.5%) and tetracycline (29.1%). The results of a study from Morocco showed that the resistance rate in raw cow’s milk was remarkably high for ampicillin (82.4%), tetracycline (70.6%), and erythromycin (23.5%) and moderate for penicillin (17.6%). As can be seen from the results of the cited authors, these acquired resistances reflect the frequency and importance of antibiotics used in veterinary medicine and animal breeding [34].

Gaglio et al. [36] found that isolates of *Enterococcus* spp. from artisanal cheeses were resistant to erythromycin (52.5%), ciprofloxacin (35.0%), and tetracycline (17.5%); this is consistent with our results only for tetracycline (18.2%). A study related to the investigation of enterococci in dairy products in Poland, including most cheeses, showed similar resistance to erythromycin and tetracycline compared to our study, but the resistance to rifampicin was lower (8.7%) [45]. Our study demonstrated resistance to vancomycin (31.8%) in isolates from cheeses; this contradicts the outcomes of other studies, in which resistance to vancomycin was very low or absent [38,45].

Our study aimed to detect only two resistance genes. In this study, *vanA* was detected in 4.41% of all enterococcal isolates. In cheeses, only 2.27% of isolates had the *vanA* gene; this is consistent with results from the study by Domingo-Lopes et al. [46], who reported that a small number (<5%) of enterococcal isolates obtained from cheeses harbored *vanA* resistance genes. Also, according to results from a similar study by Chajęcka-Wierzchowska et al. [45], the *vanA* gene was absent, even though two isolates showed phenotypic expression. Oruc et al. [47] found that 13.63% of strains of *Enterococcus* spp. isolated from traditional white cheese harbored the *vanA* gene. In our study, overall, four enterococcal isolates did not have a phenotypic manifestation of vancomycin resistance, yet the presence of one of a group of genes associated with resistance to vancomycin was demonstrated. Gaglio et al. [36] published the results of a study with a similar design to the present study (i.e., investigating milk, environment, and cheeses), but all the tested enterococci (40) were free of phenotypic resistance, and no isolates with the *vanA* gene were found. Outputs from studies investigating the presence of resistance genes in the cheesemaking environment are limited. VRE genotypes *vanA* and *vanB* are the most prevalent in Europe, and *vanA* genotypes in *E. faecium* are the most problematic for humans [8]. This contradicts our finding of the presence of the *vanA* gene only in *E. faecalis* isolates and one *E. devriesi* isolate.

Chajęcka-Wierzchowska et al. [45] showed that resistance to tetracycline in dairy products in their examination was most often conferred by *tetM* (14.3%), while in our outputs from cheeses, genotypic resistance only occurred in 6.81% of isolates. Gaglio et al. [36] described the presence of the *tetM* gene in all enterococcal isolates with resistance phenotype from milk, the environment, and cheeses. They found resistance to tetracycline in 87.5% of strains (8); in our study, the overall presence of *tetM* was 51.85% in resistant isolates (27). Meanwhile, in their study, only one isolate from milk was resistant to tetracycline in contrast to thirteen resistant isolates from raw milk in our results, in which *tetM* was detected in ten strains. In addition, our study revealed the presence of *tetM* in six isolates without phenotypic manifestation. A study in Italy published this year examined the biodiversity and antimicrobial resistance profile of enterococci in raw cow’s milk and detected the presence of the *tetM* gene in 75.76% of enterococcal isolates that were resistant to tetracycline [48]. An investigation of the presence of resistance genes in a study on raw goat’s milk showed that *tetM* was the most commonly detected resistance gene [30]. As reported by many authors, tetracycline and erythromycin resistance are widespread in the dairy environment [49,50].

As the results of our study showed, the cheesemaking environment may be the source of some resistance genes in enterococci, even though no phenotypic expression in some isolates was observed. It should also be emphasized that all swabs from the processing environment were taken after the process of cleaning and disinfection in all dairy plants. An important outcome of our study is the detection of resistance of enterococcal isolates to the reserve antibiotic rifampicin, both in the production environment and in raw milk, but especially an almost 50% resistance in cheese isolates. As our study showed, resistance at the level of phenotypic expression, but also the presence of two genes associated with AMR, does not only concern the species *E. faecium* and *E. faecalis*, but also other enterococcal species. However, the aim was to offer a complex perspective on this issue. In the future, it would be necessary to examine the relationships between the raw material, the environment, and the final product in one line, as well as to focus attention on cheeses that are made from pasteurized milk, because some undesirable bacteria can be transferred during their handling. The limitations of our study include the small number of resistance genes tested and the lack of confirmation of these genes by sequencing, which represents a stronger level of evidence for the presence of resistance genes.

## 5. Conclusions

This study showed the presence of nine enterococcal species in raw milk, the environment, and cheeses during the cheesemaking, but overall, the most common species were *E. faecalis* (38.97%) and *E. faecium* (34.56%). The phenotypic manifestation of antimicrobial resistance to some of the eight tested antibiotics in enterococcal isolates was demonstrated, regardless of whether it was raw milk, the environment of cheesemaking, or cheese made from pasteurized milk. The finding of resistance to rifampicin in all tested isolates at the level of 35.3% and in cheeses at the level of 43.2% is important due to the use of this antibiotic as a reserve in Slovakia with increasing consumption in the hospital sector. Also, the detection of the highest resistance to ampicillin in enterococcal isolates from raw milk in 56.8% is relevant, given that it is one of the most widely used antibiotics in clinical practice in Slovakia. In this study, thirty-five isolates evaluated were multidrug resistant. Overall, 20 isolates of enterococci carried *tetM*, with most of them being *E. faecalis*, and there were 5 *E. faecalis* isolates and 1 *E. devriesi* isolate with the resistance gene *vanA*. No enterococcal isolate carried both of the genes studied.

Food safety is important in every step of processing from raw materials to final product preparation for consumers. Scientists are responsible for providing a constant supply of current information regarding all areas of antimicrobial resistance, including not only human medicine but also veterinary medicine, as well as their cooperation.

## Figures and Tables

**Table 1:** The primers used in this study for the identification of *Enterococcus* spp.

Gene	Primer Name	Primer Sequence 5′-3′	Product Size (bp)	Annealing Temperature	Reference
*tuf*	tuf-F	TACTGACAAACCATTCATGATG	112	52 °C	[22]
	tuf-R	AACTTCGTCACCAACGCGAAC			

**Table 2:** The primers used in this study for the detection of resistance genes using a PCR-based method.

Gene	Primer Name	Primer Sequence 5′-3′	Product Size (bp)	Annealing Temperature	Reference
*vanA*	vanA-F	GGGAAAACGACAATTGC	723	50 °C	[28]
	vanA-R	GTACAATGCGGCCGTTA			
*tetM*	tetM-F	GTTAAATAGTGTTCTTGGAG	576	52 °C	[29]
	tetM-R	CTAAGATATGGCTCTAACAA			

**Table 3:** Enumeration of enterococci in different samples from artisanal dairy plants in Slovakia.

	Raw Milk	Produced Cheeses
Average in log_10_	3.38 CFU/mL	3.88 CFU/g
Standard deviation	±0.39	±0.79
Minimum in log_10_	2.56 CFU/mL	2.78 CFU/g
Maximum in log_10_	3.81 CFU/mL	5.48 CFU/g
Median	3.44	3.88

**Table 4:** Distribution and number of isolates among *Enterococcus* spp. identified using MALDI-TOF MS isolated from different samples associated with cheesemaking.

Species	Number of Isolates	Raw Milk	Environment	Cheeses
*E. faecalis*	53 (38.97%)	28 (63.64%)	12 (25.0%)	13 (29.55%)
*E. faecium*	47 (34.56%)	1 (2.27%)	24 (50.0%)	22 (50.0%)
*E. durans*	14 (10.29%)	4 (9.09%)	7 (14.58%)	3 (6.82%)
*E. devriesi*	8 (5.88%)	3 (6.82%)	0 (0.0%)	5 (11.36%)
*E. hirae*	5 (3.67%)	5 (11.36%)	0 (0.0%)	0 (0.0%)
*E. italicus*	4 (2.94%)	0 (0.0%)	4 (8.33%)	0 (0.0%)
*E. casseliflavus*	2 (1.47%)	1 (2.27%)	1 (2.08%)	0 (0.0%)
*E. malodoratus*	2 (1.47%)	2 (4.54%)	0 (0.0%)	0 (0.0%)
*E. gallinarum*	1 (0.74%)	0 (0.0%)	0 (0.0%)	1 (2.27%)
Total	136	44	48	44

**Table 5:** Antimicrobial-resistant phenotypes of enterococci isolated from different samples associated with cheesemaking according to EUCAST (2023).

Isolates	Resistant							
	AML	P	AMP	E	VAN	LEV	RD	TE
Total	0 (0.0%)	nda	2 (1.5%)	nda	0 (0.0%)	11 (8.1%)	nda	nda
Raw milk	0 (0.0%)	nda	2 (4.5%)	nda	0 (0.0%)	0 (0%)	nda	nda
Environment	0 (0.0%)	nda	0 (0.0%)	nda	0 (0.0%)	4 (8.1%)	nda	nda
Cheeses	0 (0.0%)	nda	0 (0.0%)	nda	0 (0.0%)	7 (15.9%)	nda	nda

nda, no data available; AML, amoxicillin; P, penicillin; AMP, ampicillin; E, erythromycin; VAN, vancomycin; LEV, levofloxacin; RD, rifampicin; TE, tetracycline.

**Table 6:** Antimicrobial-resistant phenotypes of enterococci identified by MALDI-TOF MS according to EUCAST (2023).

Isolates	Resistant							
	AML	P	AMP	E	VAN	LEV	RD	TE
*E. faecalis*	0 (0.0%)	nda	1 (1.9%)	nda	0 (0.0%)	3 (5.7%)	nda	nda
*E. faecium*	0 (0.0%)	nda	0 (0.0%)	nda	0 (0.0%)	6 (12.8%)	nda	nda
*E. devriesi*	0 (0.0%)	nda	0 (0.0%)	nda	0 (0.0%)	0 (0.0%)	nda	nda
*E. durans*	0 (0.0%)	nda	0 (0.0%)	nda	0 (0.0%)	1 (7.1%)	nda	nda
*E. hirae*	0 (0.0%)	nda	0 (0.0%)	nda	0 (0.0%)	0 (0.0%)	nda	nda
*E. malodoratus*	0 (0.0%)	nda	1 (50.0%)	nda	0 (0.0%)	0 (0.0%)	nda	nda
*E. casseliflavus*	0 (0.0%)	nda	0 (0.0%)	nda	0 (0.0%)	0 (0.0%)	nda	nda
*E. italicus*	0 (0.0%)	nda	0 (0.0%)	nda	0 (0.0%)	1 (25.0%)	nda	nda
*E. gallinarum*	0 (0.0%)	nda	0 (0.0%)	nda	0 (0.0%)	0 (0.0%)	nda	nda

*E.*, *Enterococcus*; nda, no data available; AML, amoxicillin; P, penicillin; AMP, ampicillin; E, erythromycin; VAN, vancomycin; LEV, levofloxacin; RD, rifampicin; TE, tetracycline.

**Table 7:** Antimicrobial-resistant phenotypes of enterococci isolated from different samples associated with cheesemaking according to CLSI (2023).

Isolates	Resistant							
	AML	P	AMP	E	VAN	LEV	RD	TE
Total	0 (0.0%)	19 (13.9%)	31 (22.8%)	11 (8.1%)	20 (14.7%)	4 (2.9%)	48 (35.3%)	27 (19.9%)
Raw milk	0 (0.0%)	12 (27.3%)	25 (56.8%)	1 (2.3%)	1 (2.3%)	0 (0.0%)	15 (34.1%)	13 (29.6%)
Environment	0 (0.0%)	1 (2.1%)	2 (4.2%)	2 (4.2%)	5 (10.4%)	1 (2.1%)	14 (29.2%)	6 (12.5%)
Cheeses	0 (0.0%)	6 (13.6%)	4 (9.1%)	8 (18.2%)	14 (31.8%)	3 (6.8%)	19 (43.2%)	8 (18.2%)

AML, amoxicillin; P, penicillin; AMP, ampicillin; E, erythromycin; VAN, vancomycin; LEV, levofloxacin; RD, rifampicin; TE, tetracycline.

**Table 8:** Antimicrobial-resistant phenotypes of enterococci identified by MALDI-TOF MS according to CLSI (2023).

Isolates	Resistant							
	AML	P	AMP	E	VAN	LEV	RD	TE
*E. faecalis*	0 (0.0%)	8 (15.1%)	17 (32.1%)	0 (0.0%)	18 (33.9%)	2 (3.7%)	33 (62.3%)	14 (26.4%)
*E. faecium*	0 (0.0%)	5 (10.6%)	3 (6.4%)	10 (21.3%)	0 (0%)	2 (4.3%)	7 (14.9%)	7 (14.9%)
*E. devriesi*	0 (0.0%)	0 (0.0%)	2 (25.0%)	1 (12.5%)	1 (12.5%)	0 (0.0%)	2 (25.0%)	1 (12.5%)
*E. durans*	0 (0.0%)	3 (21.4%)	3 (21.4%)	0 (0.0%)	0 (0.0%)	0 (0.0%)	1 (7.1%)	3 (21.4%)
*E. hirae*	0 (0.0%)	1 (20.0%)	3 (60.0%)	0 (0.0%)	0 (0.0%)	0 (0.0%)	3 (60.0%)	0 (0.0%)
*E. malodoratus*	0 (0.0%)	0 (0.0%)	1 (50%)	0 (0.0%)	0 (0.0%)	0 (0.0%)	0 (0.0%)	0 (0.0%)
*E. casseliflavus*	0 (0.0%)	1 (50.0%)	1 (50%)	0 (0.0%)	0 (0.0%)	0 (0.0%)	1 (50.0%)	0 (0.0%)
*E. italicus*	0 (0.0%)	0 (0.0%)	0 (0.0%)	0 (0.0%)	0 (0.0%)	0 (0.0%)	1 (25.0%)	1 (25%)
*E. gallinarum*	0 (0.0%)	1 (100%)	1 (100%)	0 (0.0%)	1 (100%)	0 (0.0%)	0 (0.0%)	1 (100%)

*E*., *Enterococcus*; AML, amoxicillin; P, penicillin; AMP, ampicillin; E, erythromycin; VAN, vancomycin; LEV, levofloxacin; RD, rifampicin; TE, tetracycline.

## Data Availability

The data presented in this study are available within the article.

## Supplementary Materials

The following supporting information can be downloaded at: https://www.mdpi.com/article/10.3390/life14070890/s1, Figure S1: agarose gel electrophoresis of PCR product of *tuf* gene title; Figure S2: agarose gel electrophoresis of PCR product of *vanA* and *tetM*; Table S1: the origin and numbers of all samples; Table S2: the origin of samples from cheesemaking environment and their frequency of enterococci.
