# Supplementary material for: Occurrence of Enterococci in the Process of Artisanal Cheesemaking and Their Antimicrobial Resistance
Source: Life (Basel). 2024 Jul 18;14(7):890. doi: 10.3390/life14070890 (PMC11277685; doi:10.3390/life14070890)
Supplement: Supplementary file 1 [file life-14-00890-s001.zip › life-3086224-supplementary.pdf]

Supplementary Materials

Figure S1. Agarose gel electrophoresis of PCR product of *tuf* gene; Lane M: size marker; Lanes 1–3: positive samples; Lanes 4–7: negative samples; Lane 8: positive control; Lane 9: negative control.

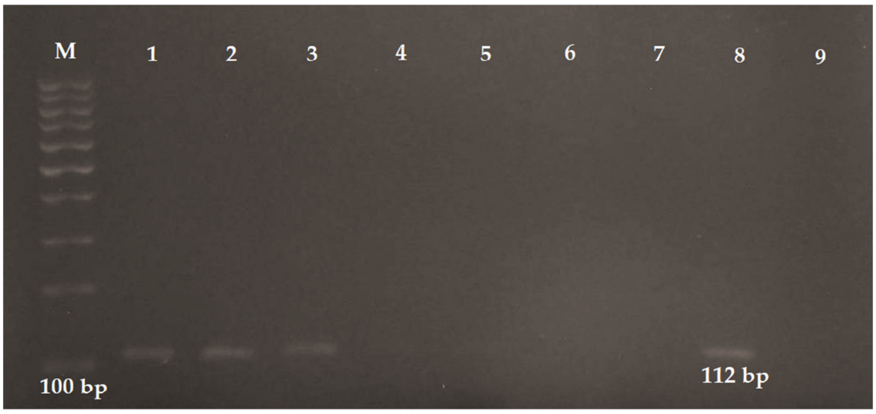

Figure S2. Agarose gel electrophoresis of PCR product of *vanA*; Lane M: size marker; Lanes 1–5: negative samples; Lanes 6 and 7: positive samples; Lane 8: positive control; Lane 9: negative control (A). Agarose gel electrophoresis of PCR product of *tetM*; Lane M: size marker; Lanes 1 and 2: positive samples; Lane 3: positive control; Lane 4: negative control (B).

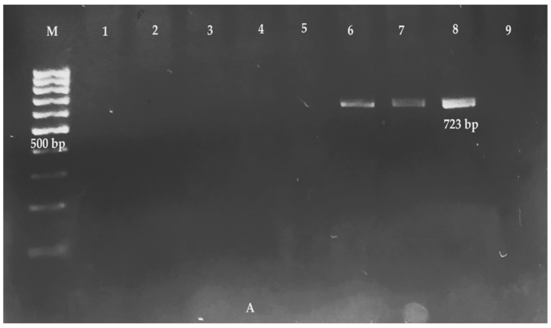

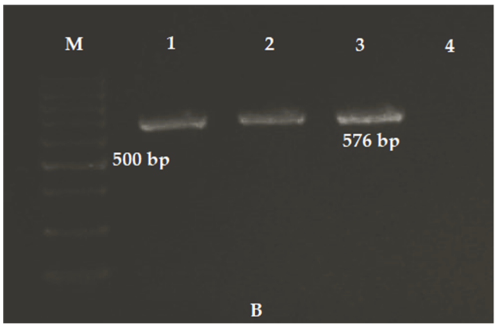

Table S1: The origin and numbers of all samples, which were taken from all dairy plants in Slovakia.

Samples	Dairy plant 1	Dairy plant 2	Dairy plant 3	Dairy plant 4
**Raw bulk milk**	2	7	2	3
**Milk after pasteurisation**	2	7	2	3
**Environment of cheesemaking**	15	13	23	24
**Cheeses**	7	6	5	6
**Total**	**26**	**33**	**32**	**36**

Table S2. The origin of samples from cheesemaking environment and number of positive samples of enterococci in four dairy plants in Slovakia**.**

	**Molds**	**Hands**	**Equipment in contact with food**	**Pasteurization equipment**	**Surrounding in rooms**	**Vats for cheesemaking**	**Tables for draining**	**Ripened rooms**	**Apron**
Total samples	**9**	**14**	**13**	**6**	**12**	**6**	**10**	**4**	**1**
Positive samples	3 (33.3%)	6 (42.8%)	13 (46.1%)	0 (0%)	3 (25%)	4 (66.7%)	4 (40%)	4 (100%)	1 (100%)
